# Maprotiline Suppresses Cholesterol Biosynthesis and Hepatocellular Carcinoma Progression Through Direct Targeting of CRABP1

**DOI:** 10.3389/fphar.2021.689767

**Published:** 2021-05-20

**Authors:** Cancan Zheng, Yidong Zhu, Qinwen Liu, Tingting Luo, Wenwen Xu

**Affiliations:** ^1^ MOE Key Laboratory of Tumor Molecular Biology and Key Laboratory of Functional Protein Research of Guangdong Higher Education Institutes, Institute of Life and Health Engineering, Jinan University, Guangzhou, China; ^2^ MOE Key Laboratory of Tumor Molecular Biology and Guangdong Provincial Key Laboratory of Bioengineering Medicine, National Engineering Research Center of Genetic Medicine, Institute of Biomedicine, College of Life Science and Technology, Jinan University, Guangzhou, China

**Keywords:** maprotiline, hepatocellular carcinoma, Cholesterol biosynthesis, MAPK/ERK signaling pathway, SREBP2, CRABP1

## Abstract

Hepatocellular carcinoma (HCC) remains one of the leading causes of cancer-related death and has a poor prognosis worldwide, thus, more effective drugs are urgently needed. In this article, a small molecule drug library composed of 1,056 approved medicines from the FDA was used to screen for anticancer drugs. The tetracyclic compound maprotiline, a highly selective noradrenergic reuptake blocker, has strong antidepressant efficacy. However, the anticancer effect of maprotiline remains unclear. Here, we investigated the anticancer potential of maprotiline in the HCC cell lines Huh7 and HepG2. We found that maprotiline not only significantly restrained cell proliferation, colony formation and metastasis *in vitro* but also exerted antitumor effects *in vivo*. In addition to the antitumor effect alone, maprotiline could also enhance the sensitivity of HCC cells to sorafenib. The depth studies revealed that maprotiline substantially decreased the phosphorylation of sterol regulatory element-binding protein 2 (SREBP2) through the ERK signaling pathway, which resulted in decreased cholesterol biosynthesis and eventually impeded HCC cell growth. Furthermore, we identified cellular retinoic acid binding protein 1 (CRABP1) as a direct target of maprotiline. In conclusion, our study provided the first evidence showing that maprotiline could attenuate cholesterol biosynthesis to inhibit the proliferation and metastasis of HCC cells through the ERK-SREBP2 signaling pathway by directly binding to CRABP1, which supports the strategy of repurposing maprotiline in the treatment of HCC.

## Introduction

According to Global Cancer Statistics in 2018, liver cancer ranks sixth and fourth in terms of incidence and mortality worldwide, with approximately 841,000 new cases and 782,000 deaths (Bray et al., 2018). Hepatocellular carcinoma (HCC), which accounts for 75–85% of liver cancer cases, is an integral type of primary liver cancer. Although the combination of surgery, radiotherapy, chemotherapy, targeted therapy and immunotherapy theoretically can produce maximum efficacy in HCC treatment, resistance might result in treatment failures and cancer relapse (Gao et al., 2017; Fu et al., 2018; Huang et al., 2018; Ruiz de Galarreta et al., 2019). Hence, it is particularly important to develop new anticancer drugs and illustrate the molecular mechanisms.

Compared with creating a new drug from scratch, repurposing approved drugs can reduce the cost and substantially shorten the research cycle. Since drug toxicology, pharmacokinetic studies and clinical trials have already been finished, these drugs with confirmed efficacy can benefit patients faster. Thus, the new usage of existing drugs has become a trend in the field of drug development, and the discovered new efficacy of approved drugs constantly expands the indications of drugs. In this paper, we hope to identify anti-HCC drugs from 1056 small molecule drugs. Maprotiline, an FDA-approved tetracyclic antidepressant, blocks the reuptake of norepinephrine by the central presynaptic membrane, which can ameliorate symptoms to achieve antidepressant function and eliminate depressed mood (Gunduz et al., 2016). However, the anticancer effect of maprotiline has never been reported. Due to the low toxicity, good tolerance and adaptability of maprotiline, it is an ideal candidate for functional repurposing. Tong et al. (2018) reported that maprotiline may help reduce the progression of pulmonary hypertension in rats, which prompted us to redefine the function of maprotiline.

Cellular cholesterol metabolism plays an important role in numerous biological activities through multifarious intermediates (Riscal et al., 2019). Therefore, cholesterol metabolism may alter cell behavior through extremely complex and diverse pathways, especially cholesterol biosynthesis, as a key link to the network of biological activities, naturally contributing to the behavior of cells (Kugel and Hingorani, 2020). Cholesterol metabolism has been proven to promote the survival and drug resistance of tumor cells to a certain extent (Silvente-Poirot and Poirot, 2014; Yan et al., 2020). We have confirmed that the AKT/ERK pathway participates in the growth and metastasis of cancer cells in our previous studies, and this pathway has been proven to be the target of many anticancer drugs (Xu et al., 2018; Hong et al., 2020; Xu et al., 2020; Hu et al., 2021). An increasing number of studies have confirmed that SREBP2 indeed plays a cancer-promoting role by regulating cholesterol metabolism in HCC (Yang et al., 2019; Che et al., 2020; Liu et al., 2020), and SREBP2 has been proven to be the target of some anticancer drugs (Kim et al., 2018; Kim et al., 2019). Cellular retinoic acid binding proteins (CRABP1 and CRABP2) are predominately located in the cytoplasm and are named for their high affinity for retinoic acid. A study indicated that all-trans retinoic acid (atRA)-CRABP1-dependent ERK1/2 activation inhibits cell growth by activating protein phosphatase 2A (PP2A) and enhancing the stability of the p27 protein in embryonic stem cells (ESCs) (Persaud et al., 2013). Conversely, in mesenchymal and neuroendocrine tumors, CRABP1 has been proven to promote tumorigenesis and metastasis (Kainov et al., 2014).

The aim of our research was to investigate the anticancer effect of the FDA-approved drug maprotiline in HCC cells and further elucidate the molecular mechanism. In this article, we demonstrated that maprotiline could restrain the growth of HCC cells through the ERK-SREBP2 signaling pathway, which suppresses cholesterol biosynthesis by interacting with CRABP1.

## Materials and Methods

### Cell Lines and Culture

The human HCC cell lines Huh7 and HepG2 were purchased from the Cell Bank of the Chinese Academy of Sciences (Shanghai, China) and cultured in DMEM (Invitrogen, Shanghai, China) with 10% fetal bovine serum (FBS; HyClone, Utah, United States) in a 37°C humidified incubator with 5% CO_2_.

### Drugs and Plasmids

Maprotiline, 5-FU (Fluorouracil), oxaliplatin and sorafenib were obtained from Selleck Chemicals (Shanghai, China). All plasmids were designed and constructed by TranSheepBio Co., Ltd. (Shanghai, China), and HCC cells were transfected with CRABP1 or vector control using Lipofectamine 3000 reagent (Invitrogen, Carlsbad, CA, United States) according to the manufacturer’s instructions.

### Cell Viability Assay

Cells were digested and plated into each well of a 96-well plate after adjusting the cell suspension to the appropriate concentration of 1×10^3^ cells per 50 μL. After the corresponding time of cultivation, CCK-8 assays (Dojindo, Japan) were used to measure the absorbance of the well to reflect cell growth by using the microplate spectrophotometer (BioTek Instruments, Winooski, VT, United States).

### Colony Formation Assay

Cells were seeded in the wells of 6-well culture plates with 2 mL of complete DMEM, replacing the previous medium with new complete DMEM containing different concentrations of maprotiline on the second day. After two weeks of subculture, the colonies were fixed with methanol and stained with crystal violet. Finally, the cell clones were dried and photographed at ×100 magnification.

### Migration and Invasion Assays

The migration assay was performed as described previously (Xu et al., 2021). Cells were seeded in the matched upper chambers with serum-free DMEM, where DMEM supplemented with 20% FBS was added to the bottom chamber. Then, the chambers were incubated in a 37°C incubator for 24 h and detected similarly to the colony formation assay. For the invasion assay, Matrigel (Corning, United States) was diluted in FBS-free DMEM at a 1:25 ratio. The following steps were the same as the cell migration assay.

### Apoptosis Assay

Cells were gently collected with EDTA-free trypsin, and re-suspended with binding buffer. Under light-proof conditions, Annexin Ⅴ-FITC and Propidium Iodide (PI) (KeyGen, Nanjing, China) were added to the cell suspension and mixed, followed by incubation for 5–15 min at room temperature to ensure dye binding. The number of apoptotic cells was determined by flow cytometry with a FACSCalibur system (BD Biosciences) within 1 h.

### Western Blot Analysis

The total cell lysates were extracted by RIPA lysis buffer (Beyotime, Shanghai, China), and BCA protein assay kit (Thermo Scientific) was used to determine protein concentration. The proteins were separated by sodium dodecyl sulfate salt-polyacrylamide gel electrophoresis (SDS-PAGE) and transferred to a polyvinylidene difluoride (PVDF) membrane. After blocking in 5% nonfat dried milk for 2 h, washing the membrane to remove the milk. The primary antibody was incubated overnight at 4°C. The membrane was washed five times for 10 min each time in TBST, after which HRP-labeled secondary antibody was added to the membrane for 1 h at room temperature. The washing operation was repeated, and the reaction was assessed using chemiluminescence (ECL) western blotting kit. The primary antibodies for EMT, cleaved PARP, cleaved caspase-6, cleaved caspase-9, p-ERK, ERK, SREBP2, p-SREBP2, CRABP1, and β-actin and secondary antibodies were purchased from Cell Signaling Technology (Massachusetts, United States).

### RNA Sequencing and Ingenuity Pathway Analysis

The extraction of total RNA was carried out by using the HiPure Total RNA Mini Kit (Megen, Guanzhou, China). RNA sequencing (RNA-seq) was performed by the Beijing Genomics Institute Tech (Shenzhen, Guangdong, China). The gene expression in the treatment group was more than 1.5 times that of the control group, and these genes were defined as differentially expressed. IPA software (Ingenuity Systems, Redwood City, CA, United States) was used to search for potential targeted pathways. The Raw data of the project were deposited at SRA database with the access number SRR14191074 for non-treatment, and SRR14191072 for maprotiline-treatment.

### Subcutaneous Xenografts in Nude Mice

The use and handling of animals were reviewed and approved by the Ethics Committee for Animal Experiments of Jinan University. All experiments were approved by the Animal Research and Use Committee of Jinan University. The details were the same as those of the tumor xenograft experiments presented in our previous studies (Li et al., 2017). Nude mice (BALB/C nu/nu, 4–6 weeks old, female) reared under standardized conditions were purchased from GemPharmatech Co., Ltd. (Nanjing, Jiangsu, China). Huh7 cells in equal volumes of PBS and Matrigel were implanted subcutaneously into the flanks of nude mice. When the volume of the tumors reached approximately 50 mm^3^, the mice were randomly divided into three groups (*n* = 5) and treated with intraperitoneal injection of either PBS or maprotiline (20 mg/kg, 40 mg/kg) twice a week for three weeks. The mice were sacrificed after the treatment course, the tumors and blood for further tests were collected and properly stored. Cholesterol, alanine aminotransferase (ALT), aspartate aminotransferase (AST), red blood cells (RBCs), white blood cells (WBCs), neutrophils, lymphocytes, hemoglobin (HGB) and platelets (PLTs) were measured by Servicebio Biotechnology Company (Wuhan, China).

### Molecular Docking

Potential targets of the compound maprotiline were determined using the Inverse Docking protocol in the Yinfo Cloud Platform (https://cloud.yinfotek.com/). A virtual screening library of protein targets was built based on the sc-PDB (Kellenberger et al., 2006) and UniProt databases. The AutoDock Vina (Trott and Olson, 2010) program was employed to dock the compound into the pocket of each protein, and then, the RDKit's Shape Tanimoto distances (RDKit, Open-Source Cheminformatics. http://www.rdkit.org) were calculated to compare the docked poses with the crystal ligand. Finally, a set of top ranked potential targets was selected.

### Purification of CRABP1 Protein

Human CRABP1 cDNA was inserted into pET-28a (+) by TranSheepBio Co., Ltd., with a His-tag. The plasmid was transformed into BL21 competent cells, and then, a single colony containing the pET-28a (+)/CRABP1 plasmid was picked and cultured at a temperature of 25°C and a rotation speed of 180 rpm/min for 6 h. IPTG was added to the bacterial fluid at a concentration of 0.5 mM and cultured overnight. The cells were collected by centrifugation, washed twice with deionized water and suspended in PBS, after which the target protein was harvested by ultrapressure lysis and centrifugation. The protein was purified by a Ni2-Sepharose plate column, eluted in 250 M gradient imidazole buffer and eluted completely in PBS (pH 7.4) buffer. The concentration of purified protein was determined by a Nanodrop 2000/2000c system.

### Surface Plasmon Resonance (SPR) Analysis

To confirm the binding of the CRABP1 protein and maprotiline, we carried out surface plasmon resonance analysis on a Biacore T100 biosensor system (GE Healthcare Life Sciences, United States). CRABP1 was diluted to 10 mM with acetate (pH 5.0) and immobilized onto an active CM7 chip (GE Healthcare Life Sciences, United States) as described previously (Xu et al., 2020). The maprotiline was diluted with 1% PBS buffer containing 0.5% Tween-20 (pH 7.4) at different concentrations and then flowed through the CM7 chip at a flow rate of 30 μL min ^−1^ for 600 s for binding. Oxaliplatin and 5-FU were used as the negative controls. The combination was analyzed by admin@Biacore X100 Evaluation Software.

### Statistical Analysis

All experiments were independently performed three times, and the data are presented as the mean ± SD. All statistical analyses and figures were performed by using GraphPad Prism software (San Diego, CA, United States) with Student’s *t* test. For all analyses in this article, statistical significance was defined as *p < 0.05, **p < 0.01, ***p < 0.001, and ****p < 0.0001.

## Results

### Maprotiline Induces Apoptosis and Inhibits Cell Growth in HCC Cells

**FIGURE 1:**
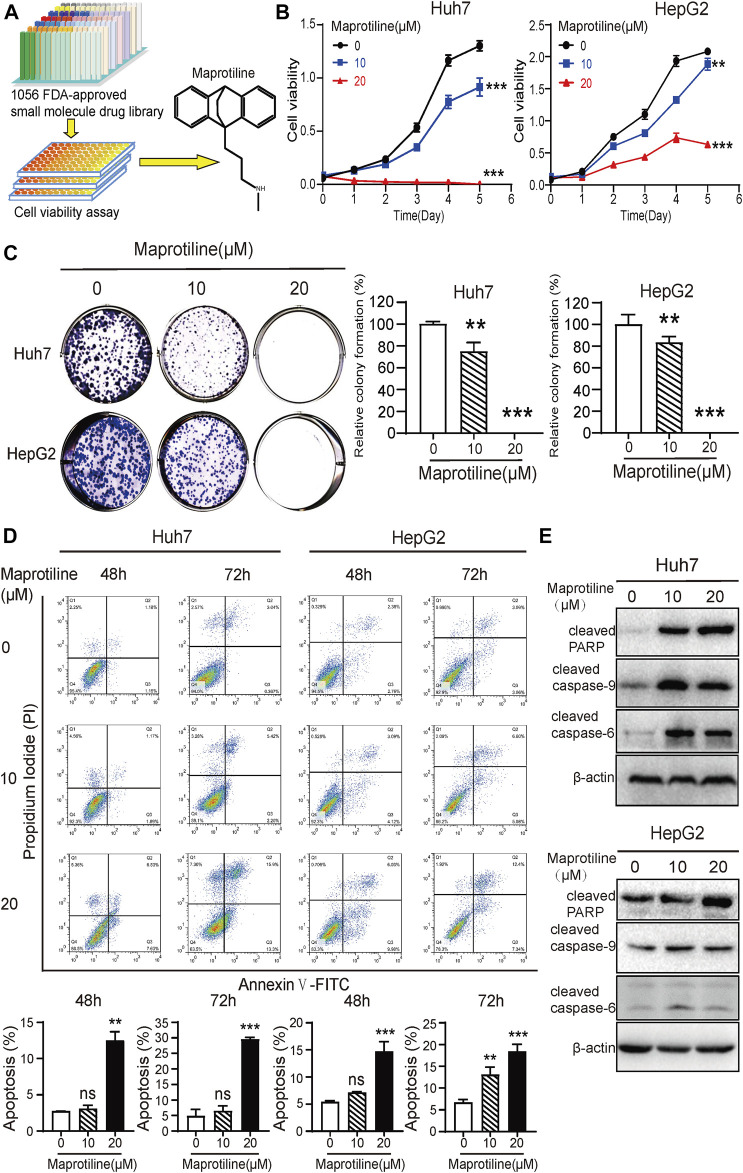
Maprotiline inhibits growth and induces apoptosis in Huh7 and HepG2 HCC cells. **(A)** The drug screening procedure and the molecular structure of maprotiline. **(B)** Cell viability assays of Huh7 and HepG2 cells treated with 0, 10, or 20 μM maprotiline for 0, 24, 48, 72, 96, or 120 h. **(C)** Maprotiline inhibited the colony formation ability of Huh7 and HepG2 cells. **(D)** The detection of apoptosis in Huh7 and HepG2 cells treated with maprotiline at concentrations of 0, 10, and 20 μM for 48 and 72 h by an Annexin V-FITC/PI double staining assay. **(E)** Western blots of cleaved caspase-6, cleaved caspase-9 and cleaved caspase-PARP in Huh7 and HepG2 cells treated with 0, 10, or 20 μM maprotiline for 72 h.

Based on a drug library consisting of 1056 FDA-approved small molecules, cell viability assays were performed to identify drugs with anti-proliferative effects on HCC cells. After several rounds of large-scale experimental screening, we finally confirmed that maprotiline, which has never been previously reported to have anticancer function, has a good killing effect on HCC cells (Figure 1A). In this research, we wanted to further explore the anticancer effect and mechanism of maprotiline. Maprotiline, as a representative tetracycline antidepressant, has been widely applied in the clinic due to its few side effects with good adaptability and tolerance, indicating that its repurposing will be advantageous. As shown in Figure 1B, maprotiline markedly inhibited the cell viability of Huh7 and HepG2 cells in a dose- and time-dependent manner. Next, we conducted colony formation assays of Huh7 and HepG2 cells to further investigate the long-term effect of maprotiline on HCC cells (Figure 1C). As expected, the size and number of colonies were significantly reduced with the increasing maprotiline concentration, and when the maprotiline concentration reached 20 μM, no cells survived. Moreover, apoptosis assay results showed that maprotiline could induce cell apoptosis in Huh7 and HepG2 cells (Figure 1D), which partly explains the cytotoxicity of maprotiline to HCC cells. The induction of apoptosis in Huh7 and HepG2 cells by maprotiline was further shown by the increase of apoptotic proteins, including cleaved caspase-6, cleaved caspase-9 and cleaved poly ADP ribose polymerase (PARP), as shown in Figure 1E. Taken together, these data confirmed that maprotiline can trigger apoptosis to restrain the proliferation of HCC cells.

### Maprotiline Represses the Metastasis of HCC Cells *In Vitro*

**FIGURE 2:**
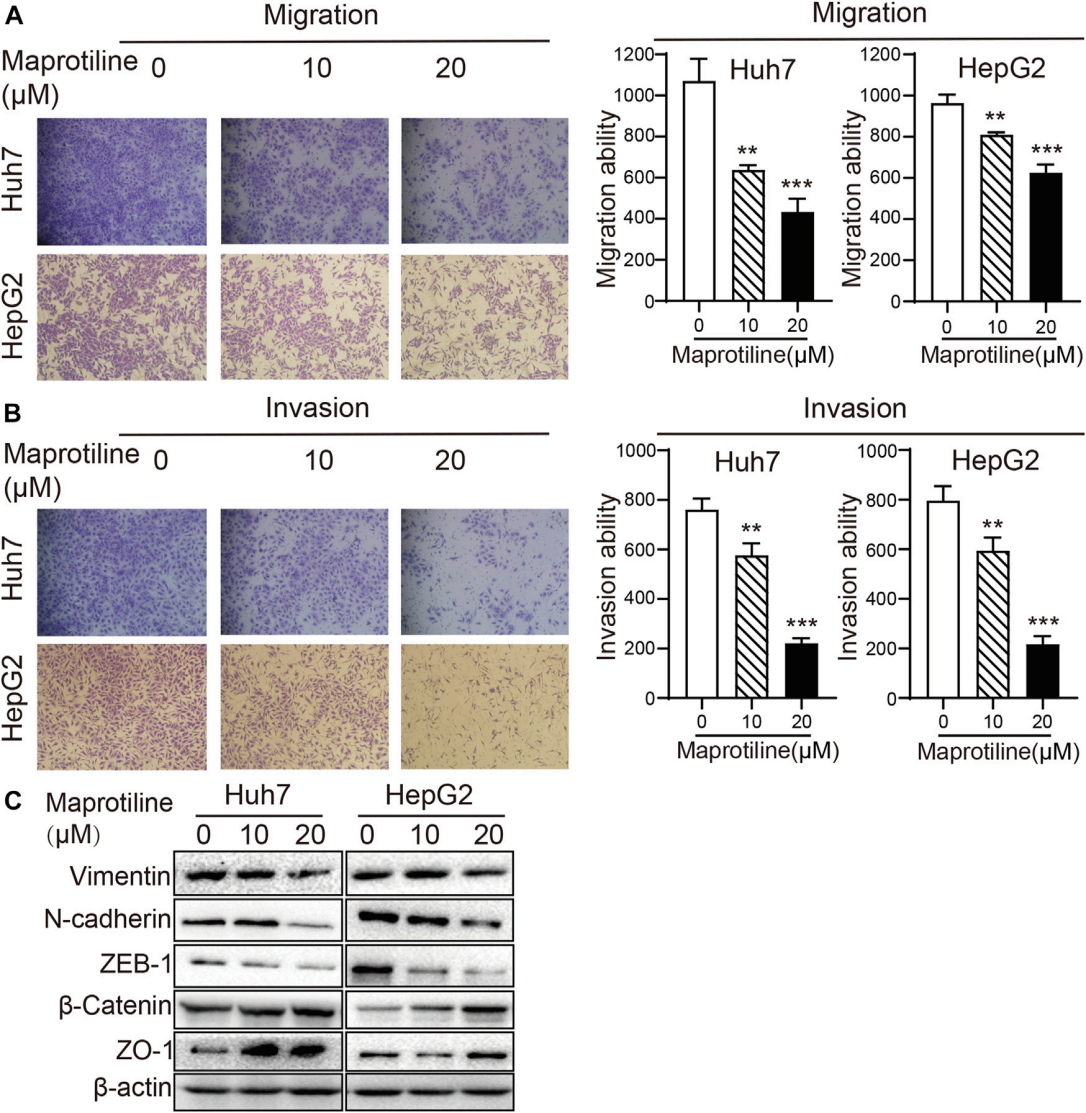
Maprotiline suppresses the migration and invasion of Huh7 and HepG2 HCC cells. **(A, B)** The migration and invasion of Huh7 and HepG2 cells were substantially inhibited by treatment with maprotiline at concentrations of 10 and 20 μM for 24 h, and wells without maprotiline were used as controls. **(C)** Huh7 and HepG2 cells were exposed to maprotiline at concentrations of 0, 10, and 20 μM for 72 h. Expression of the EMT-related proteins ZO-1, β-catenin, ZEB-1, N-cadherin and Vimentin displayed a dose-dependent effect in Western blot results, and β-actin was used as an internal reference protein.

Next, to explore the effect of maprotiline on the invasion of HCC cells, we conducted Transwell assays in Huh7 and HepG2 cells. Cell migration assays indicated that maprotiline significantly restrained HCC cells migration (Figure 2A); moreover, the outcome of the invasion experiments was similar (Figure 2B). As such, we carried out Western blotting to further detect whether the expression of epithelial-mesenchymal transition (EMT)-related proteins relevant to tumor metastasis changed. Western blot results showed that maprotiline inhibited EMT, as indicated by the upregulation of ZO-1 and β-catenin expression as well as the downregulation of ZEB-1, N-cadherin and vimentin expression in a dose-dependent manner (Figure 2C). Here, we found that maprotiline could repress the metastatic ability of Huh7 and HepG2 cells by weakening EMT.

### Maprotiline Enhances the Sensitivity of HCC Cells to Sorafenib

**FIGURE 3:**
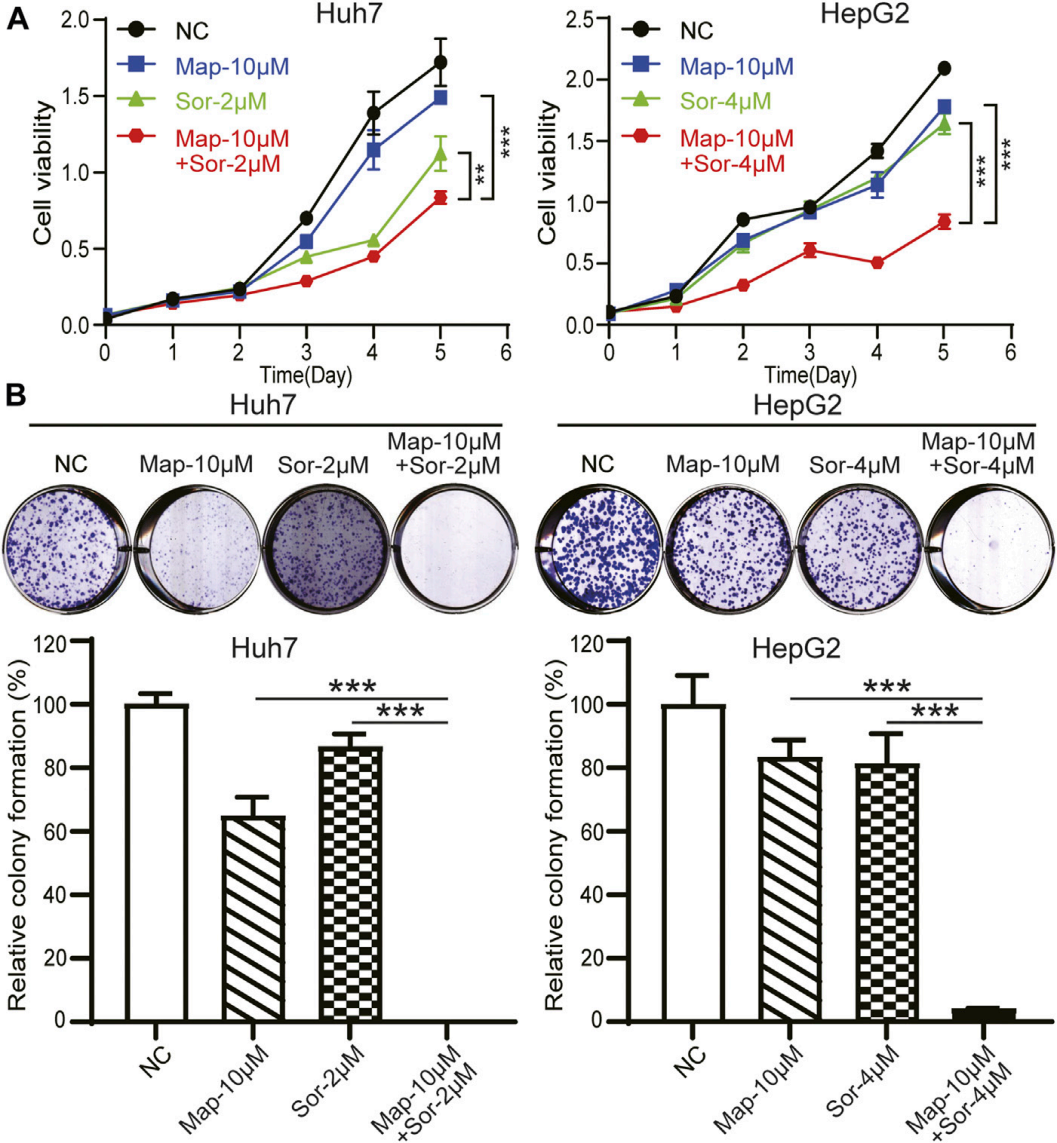
Maprotiline in combination with sorafenib inhibits the growth of Huh7 and HepG2 HCC cells. **(A)** Cell viability assays of Huh7 and HepG2 cells exposed to maprotiline alone or in combination with sorafenib for 0, 24, 48, 72, 96, and 120 h. **(B)** Colony formation assays of Huh7 and HepG2 cells under 10 μM maprotiline treatment alone or with sorafenib.

Sorafenib is the standard treatment in advanced hepatocellular carcinoma, however, chemotherapy resistance remains a crucial challenge for patients (Gao et al., 2017; Mir et al., 2017; Lu et al., 2018; Mahipal et al., 2019; Wai Ling Khoo et al., 2019; Xiang et al., 2019). To explore the biological function of maprotiline in hepatocellular carcinoma chemoresistance, we detected the sensitivity of Huh7 and HepG2 cells to sorafenib in the presence or absence of maprotiline by cell viability assays and colony formation assays. The combination of low-dose maprotiline and low-dose sorafenib markedly inhibited the growth (Figure 3A) and colony forming ability of HCC cells (Figure 3B) compared to low-dose maprotiline or sorafenib alone. Collectively, these experiments indicated that maprotiline can enhance the sensitivity of HCC cells to sorafenib.

### Maprotiline Inhibits Cholesterol Biosynthesis in HCC Cells Through the MAPK/ERK Pathway

**FIGURE 4:**
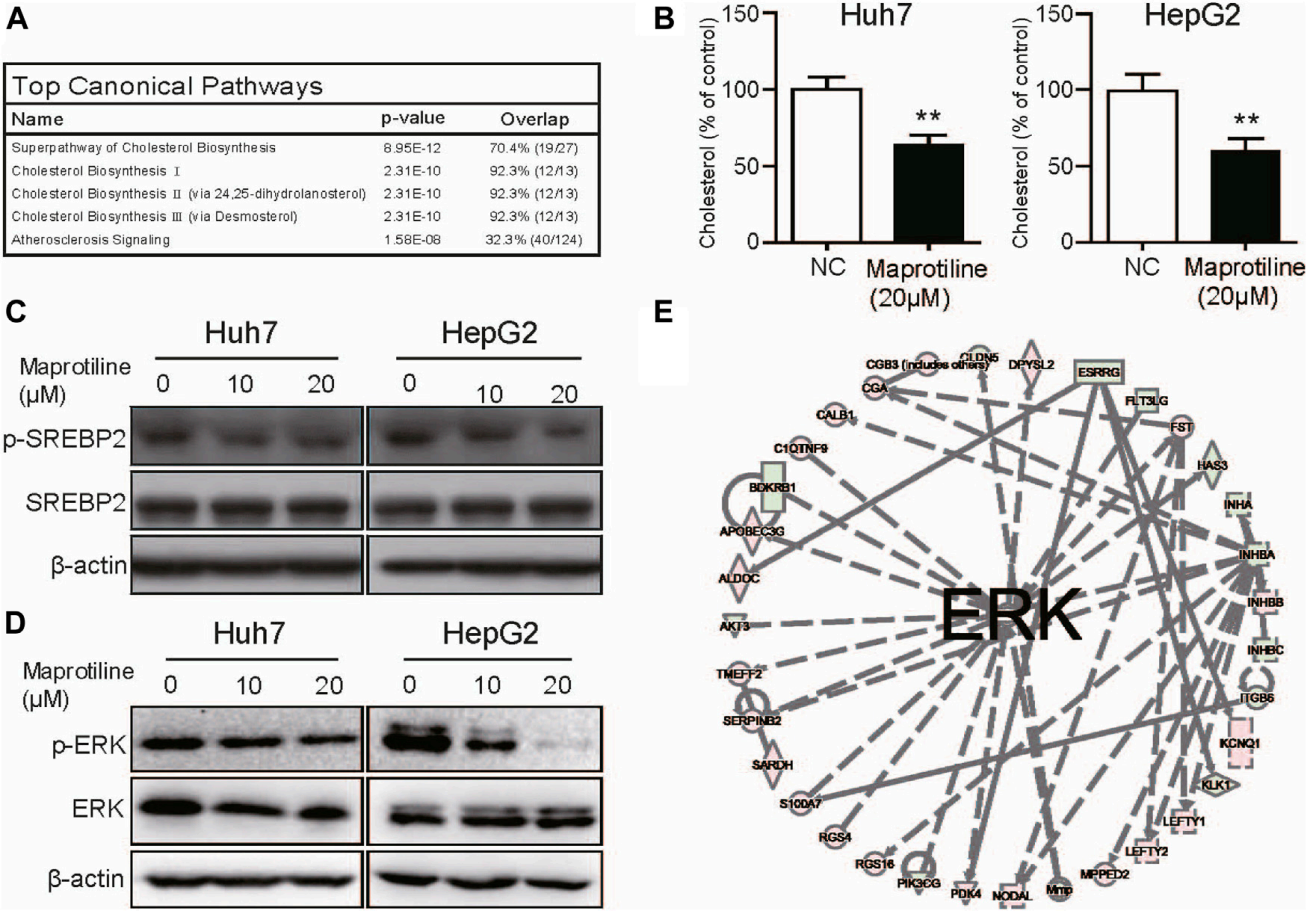
Maprotiline arrests cholesterol biosynthesis in HCC cells through the ERK pathway. **(A)** Top 5 RNA-seq pathways in enrichment analysis. **(B)** Cholesterol levels in the maprotiline-treated HepG2 and Huh7 cells. **(C)** Ingenuity pathway analysis (IPA) reflected dysregulation of the ERK pathway in the maprotiline-treated HepG2 cells. **(D)** Western blot of phosphorylated SREBP2 in the maprotiline-treated HepG2 and Huh7 cells. **(E)** HepG2 and Huh7 cells were cultured with 0, 10, or 20 μM maprotiline for 72 h, and ERK and p-ERK were detected by Western blots.

Then, to investigate the molecular mechanism by which maprotiline inhibits tumor growth and metastasis in HCC, we conducted RNA sequencing to compare gene expression profiles between 20 μM maprotiline-treated HepG2 cells and nontreated HepG2 cells. The results indicated that there was a significant difference in the cholesterol biosynthesis pathway between the maprotiline-treated HepG2 cells and the negative control cells, suggesting that maprotiline possibly affect the survival of HCC cells via cholesterol metabolism (Figure 4A). Based on our hypothesis, we tested the cholesterol level of Huh7 and HepG2 cells in the presence or absence of maprotiline (Figure 4B). The cholesterol level of the drug treatment group was significantly lower than that of the control group in both cell lines. Moreover, ingenuity pathway analysis (IPA) suggested dysregulation of the ERK pathway in the maprotiline-treated HepG2 cells (Figure 4C). To further confirm the regulation of the ERK pathway by maprotiline, we examined the expression of ERK pathway associated proteins. As shown in Figure 4D, p-ERK showed a concentration-dependent decrease after maprotiline treatment, which demonstrated that maprotiline suppresses the activation of the ERK pathway. SREBP2 has been reported to regulate cholesterol metabolism downstream of the ERK pathway, which naturally attracted our attention and interest. Consequently, Western blotting was conducted to probe whether SREBP2 contributes to the effect of maprotiline in Huh7 and HepG2 cells, and our results confirmed that maprotiline might restrain cholesterol biosynthesis by inhibiting SREBP2 phosphorylation (Figure 4E). These findings revealed that maprotiline probably attenuate cholesterol biosynthesis in HCC cells through the ERK pathway and subsequent phosphorylation of SREBP2.

### Maprotiline Directly Targets CRABP1 in HCC Cells

**FIGURE 5:**
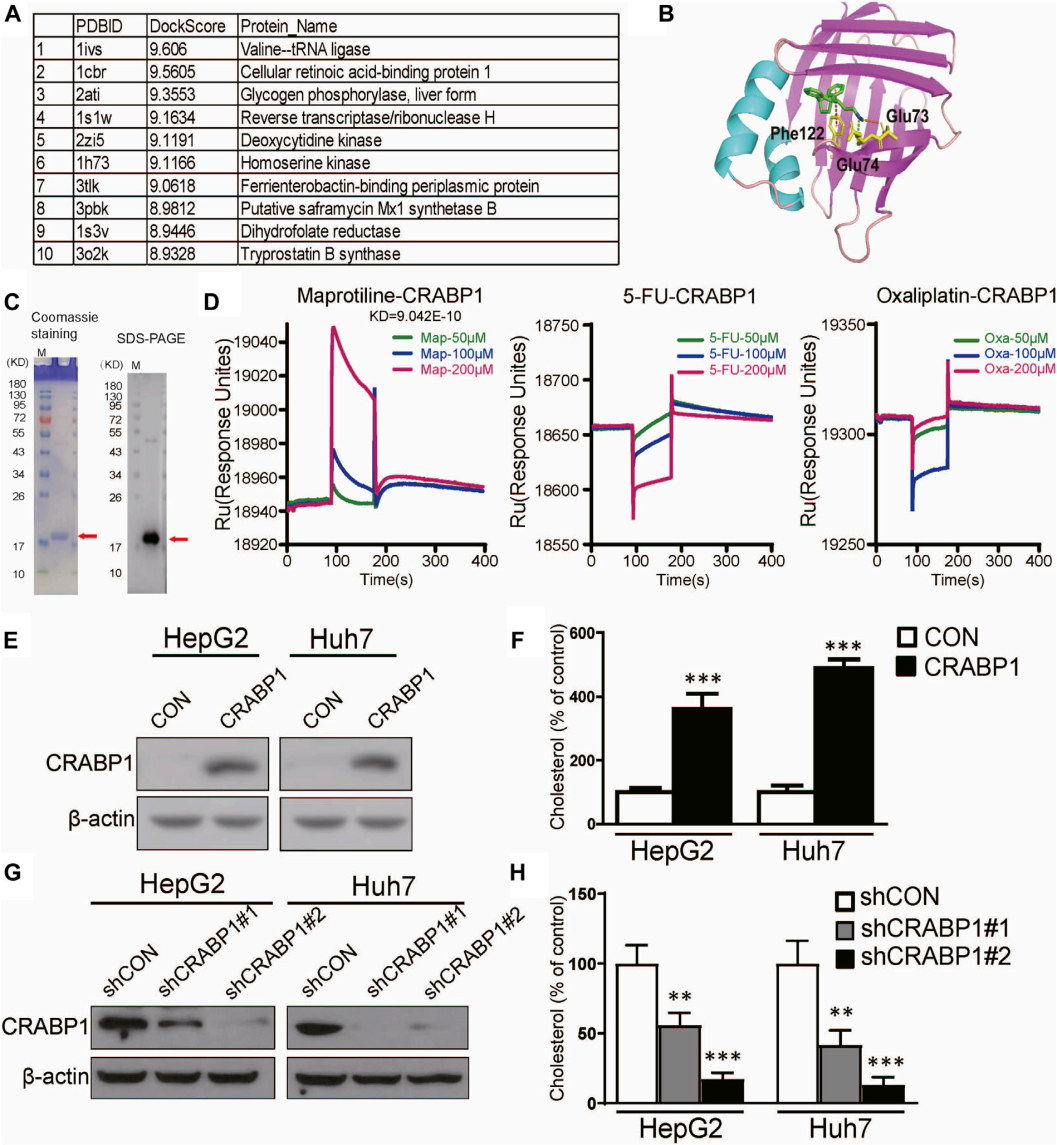
Maprotiline directly targets CRABP1 in HCC cells. **(A)** Top 10 molecular docking scores. **(B)** Molecular docking of maprotiline and CRABP1. **(C)** Coomassie staining and Western blot of purified CRABP1 protein; the red arrow indicates the CRABP1 band. **(D)** SPR analysis revealed the binding between maprotiline and the CRABP1 protein. 5-FU and oxaliplatin were used as negative controls. **(E)** The overexpression of CRABP1 in HCC cells. **(F)** The relative cholesterol level of the CRABP1-overexpressing HCC cells. **(G)** The knockdown of CRABP1 in HCC cells. **(H)** The relative cholesterol level of the CRABP1 knockdown HCC cells.

To determine the target of maprotiline in HCC cells, we used molecular docking to search the potential target proteins (Figure 5A). Among the potential target proteins screened by molecular docking with the maprotiline molecular structure (Figure 5B), CRABP1 was selected based on existing research. We successfully expressed and purified CRABP1 (Figure 5C). Then, we verified the binding of maprotiline and CRABP1 by the surface plasmon resonance (SPR) assay. The results from the SPR assay confirmed that maprotiline could bind to CRABP1 *in vitro*, which suggested that maprotiline probably produces anticancer effects by directly targeting CRABP1 (Figure 5D). Subsequently, we aimed to verify the cancer-promoting effect of CRABP1 at the cellular level, so we constructed CRABP1-overexpressing cell lines (Figure 5E). We found that overexpression of CRABP1 could improve cholesterol levels in HCC cells (Figure 5F), which confirmed that CRABP1 can promote HCC cell proliferation. In addition, knockdown of CRABP1 significantly reduced cholesterol levels compared with those of the wild-type cells (Figures 5G,H), which further supports our conclusion. Here, we provide the first evidence showing that CRABP1 may be the target of maprotiline and that regulate cholesterol biosynthesis.

### Maprotiline Suppresses the Growth of HCC Tumor Xenografts *In Vivo*

**FIGURE 6:**
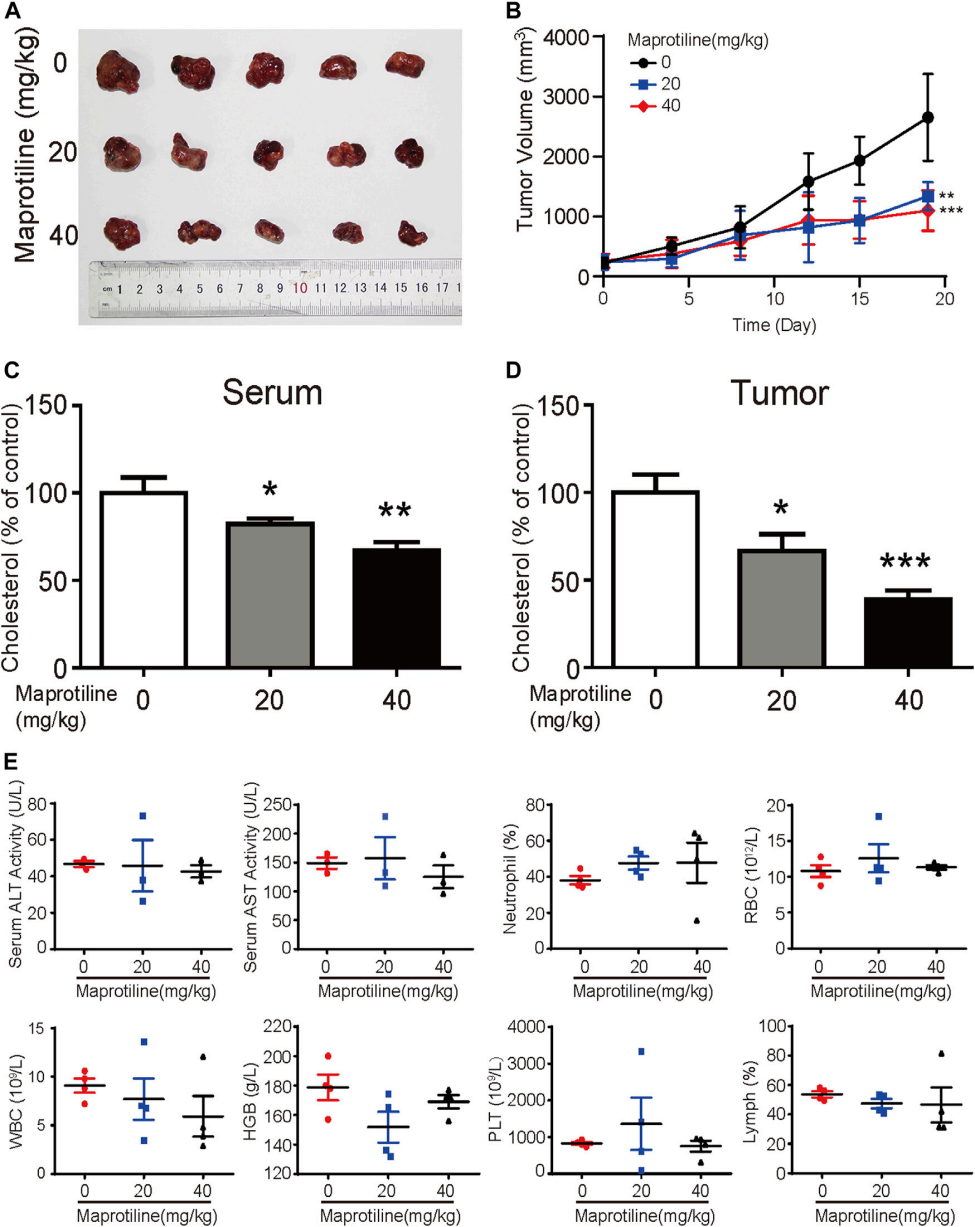
Maprotiline suppresses the growth of HCC tumor xenografts *in vivo*. **(A)** Image of tumors displaying significant growth inhibition of Huh7-derived tumor xenografts (*n* = 5) by maprotiline. **(B)** The tumor growth curve shows that maprotiline significantly suppressed the growth of tumor xenografts. **(C)** The relative cholesterol level in the serum of the maprotiline-treated nude mice compared with the control mice. **(D)** The relative cholesterol level in the tumors of the maprotiline-treated nude mice compared with that of the control group. **(E)** Comparison of serum alanine aminotransferase (ALT) and aspartate aminotransferase (AST) levels between the maprotiline-treated and control groups, as well as red blood cells (RBCs), white blood cells (WBCs), neutrophils, lymphocytes, hemoglobin (HGB) and platelets (PLTs).

Based on our *in vitro* experiments, we further explored the antitumor effect of maprotiline on HCC cells *in vivo.* Huh7 cells were implanted subcutaneously into the flanks of nude mice. When the tumor volume reached approximately 50 mm^3^, the mice were randomly divided into three groups. Over the next three weeks, these mice with hepatocellular carcinoma cells were treated with PBS or maprotiline (20 mg/kg, 40 mg/kg) through intraperitoneal injection twice a week. As shown in Figures 6A,B, maprotiline obviously suppressed the growth of Huh7-derived tumor xenografts compared to that of the control group. On the basis of the analysis of tumors and serum in nude mice, we found that the cholesterol levels in serum and tumors of the maprotiline-treated groups show a dose-dependent decrease compared with those of the control groups (Figures 6C,D). This finding proves that maprotiline may inhibit the growth of tumors by weakening the biosynthesis of cholesterol. To confirm whether maprotiline can produce strong toxicity and side effects on the organs, immune system and hematopoietic function, we examined the organs and blood of mice. Our results indicated that maprotiline treatment did not result in a significant change in the serum alanine transaminase (ALT) and aspartate transaminase (AST) levels of nude mice or changes in red blood cells (RBCs), white blood cells (WBCs), neutrophils, hemoglobin (HGB) and platelets (PLTs), suggesting that maprotiline had no obvious toxic effect on animals (Figure 6E). Thus, these data demonstrated the treatment efficiency and safety of maprotiline as an anticancer agent.

## Discussion

**FIGURE 7:**
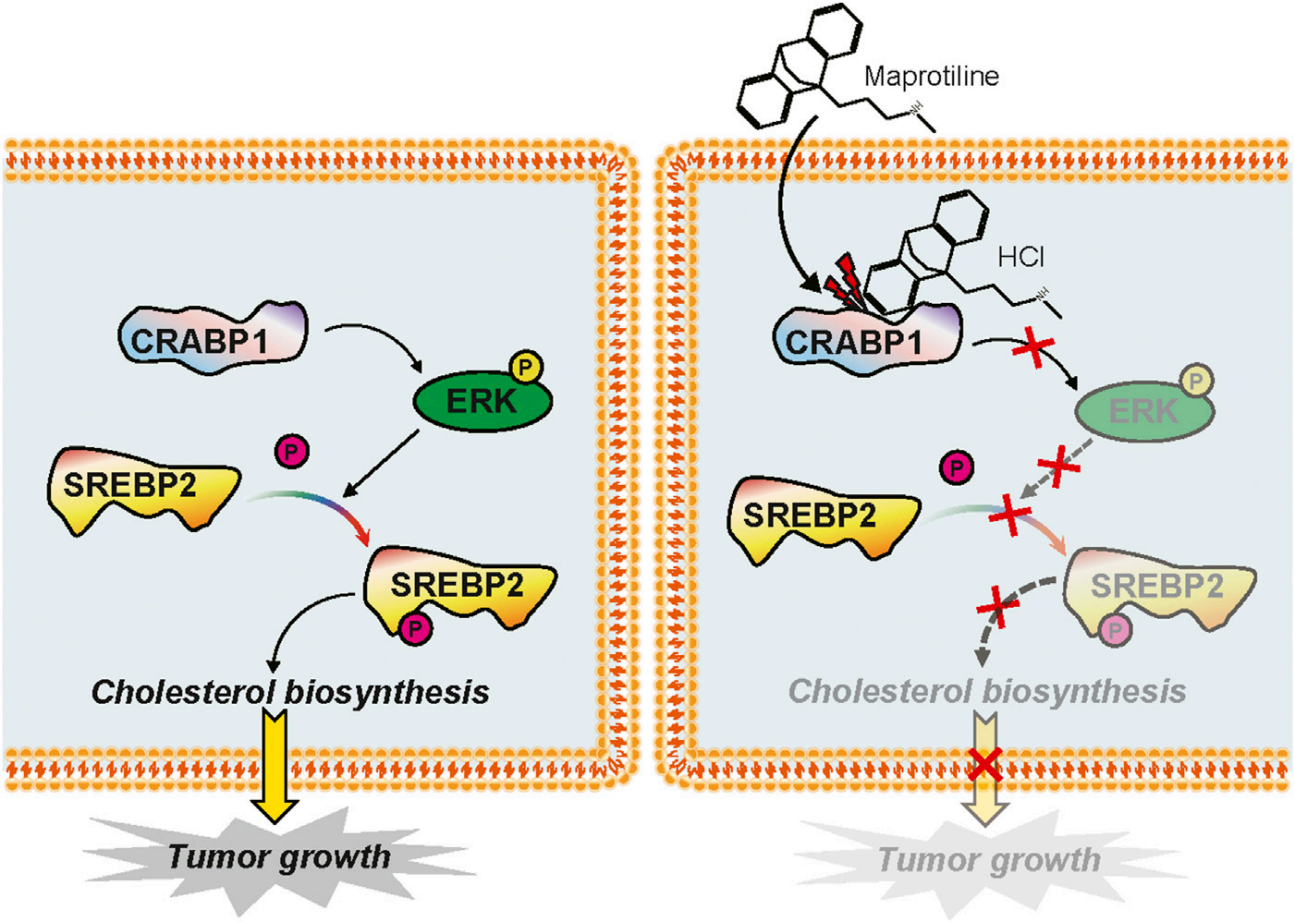
Mechanism diagram of maprotiline in HCC cells.

Maprotiline has been recognized as an antidepressant for a long time, however, several studies have reported its anti-inflammatory function (Rafiee et al., 2016) and ability to relieve pulmonary hypertension (Tong et al., 2018). In this paper, we pointed out that maprotiline has an anticancer effect on HCC. First, we found that maprotiline could inhibit the growth of Huh7 and HepG2 cells in a dose- and time-dependent manner and induce apoptosis (Figure 1). Next, we further revealed that maprotiline inhibited cell migration, invasion and EMT phenotypes (Figure 2). The EMT phenotype is always involved in a variety of cancer cell resistance mechanisms, thus, maprotiline may be able to kill drug-resistant cells. In addition, the combination of drug treatments proved that maprotiline can enhance the efficacy of sorafenib (Figure 3), which also indicates that maprotiline could be an important supplement to prevent sorafenib resistance. Then, we pointed out maprotiline could decrease SREBP2 phosphorylation by RNA-Seq combined with IPA, which possibly result in a decrease in cholesterol biosynthesis through the ERK pathway (Figure 4). Since the antitumor effect and mechanism of maprotiline have never been reported before, we sought to identify the targets of maprotiline by molecular docking. We successfully found and verified for the first time that CRABP1 may be the target of maprotiline in HCC cells (Figure 5). Except for the discovery and verification of the antitumor activity and molecular mechanism of maprotiline, we also confirmed the efficacy and biological safety of maprotiline *in vivo* through subcutaneous xenografts in nude mice (Figure 6). Thus, we can roughly deduce the anticancer mechanism of maprotiline in HCC cells (Figure 7). In HCC cells, CRABP1 probably promote the phosphorylation of SREBP2 by activating the ERK pathway. Subsequently, as an important regulatory element of cholesterol biosynthesis, phosphorylated SREBP2 may promote cholesterol biosynthesis as well as tumor growth. Maprotiline can directly bind to CRABP1 and inhibit its biological activity, which possibly suppress the phosphorylation of SREBP2 caused by ERK pathway activation and finally inhibit cholesterol biosynthesis and tumor growth. In conclusion, we verified the anticancer effect of maprotiline *in vitro* and *in vivo*, which supports the possibility of applying maprotiline as an anticancer drug.

Maprotiline has been reported to induce the type II cell death (autophagy) in Burkitt lymphoma, which provides a basis for us to study the anticancer function of this drug. In terms of mechanism, it has been reported that maprotiline can activate the JNK-related caspase-3 pathway to induce apoptosis, which provides a reference for the apoptosis induced by maprotiline in our study. We further confirmed that maprotiline can reduce the level of phosphorylated ERK in treated HCC cells and weaken signal transduction. The roles of the ERK pathway have been widely reported in the process of cancer development. In our previous studies, the ERK pathway was confirmed to promote tumor growth, yet the effects of maprotiline on ERK pathways have not been reported. In this paper, we revealed that the ERK pathway probably affect cholesterol synthesis through SREBP2. SREBP2 has been proven to be one of the important regulatory elements of cholesterol metabolism in many studies, and cholesterol metabolism mediated by SREBP2 has been proven to have a tumor-promoting effect. Many studies have shown that CRABP1 is closely related to the ERK pathway, suggesting we should focus on the role of CRABP1 when exploring the molecular mechanism of maprotiline's anticancer effect (Persaud et al., 2016; Lin et al., 2017; Park et al., 2019). In previous studies, maprotiline inhibited the HERG channel in the human heart through the drug binding site Y652 (Ferrer-Villada et al., 2006). Here, we demonstrated that CRABP1 may be the direct target of maprotiline in HCC, which will provide a reference for the further study of CRABP1 and maprotiline. Due to the complexity of cell activity, we didn’t exclude the possibilities that maprotiline may have other targets to suppress HCC cells besides what we mentioned above. The new targets and molecular mechanisms will be our future research interest.

The major obstacle of chemotherapy is drug resistance, and this condition continues to worsen. The molecular mechanisms of tumor resistance are extraordinarily complex, and the drug resistance mechanisms of different drugs are different, even the same drug has various drug resistance mechanisms. At present, it is difficult to reverse chemotherapy resistance, thus, finding new drugs for effective treatment of tumors may be a good choice. The existing FDA drugs have been proven to have pharmacokinetic characteristics and safety. Reinterpretation of drug efficacy will avoid the high clinical failure rate caused by ADMET, which reduces the R&D cost and shortens the time of manufacture. Celecoxib was initially approved by the Food and Drug Administration (FDA) as an analgesic, anti-inflammatory and antipyretic drug, followed by a large amount of preclinical evidence showing that celecoxib has a strong killing effect on cancer (Li et al., 2018; Zuo et al., 2018; Liu et al., 2019; Zhang et al., 2020). Therefore, the anticancer activity of maprotiline will be promising for development as a new kind of HCC treatment drug.

In general, we first verified the antitumor function of maprotiline *in vitro* and *in vivo* through cell experiments and nude mouse experiments. Mechanistically, we proposed that maprotiline may restrain cholesterol biosynthesis to inhibit the growth and metastasis of HCC cells through the ERK-SREBP2 signaling pathway by interacting with CRABP1. These results will support maprotiline as a candidate anticancer drug.

## Data Availability

The raw data supporting the conclusions of this article will be made available by the authors, without undue reservation.

## References

[B1] Bray F. Ferlay J. Soerjomataram I. Siegel R. L. Torre L. A. Jemal A. (2018). Global Cancer Statistics 2018: GLOBOCAN Estimates of Incidence and Mortality Worldwide for 36 Cancers in 185 Countries. CA. Cancer J. Clinicians. 68 (6), 394–424. 10.3322/caac.21492 30207593

[B2] Che L. Chi W. Qiao Y. Zhang J. Song X. Liu Y. (2020). Cholesterol Biosynthesis Supports the Growth of Hepatocarcinoma Lesions Depleted of Fatty Acid Synthase in Mice and Humans. Gut 69 (1), 177–186. 10.1136/gutjnl-2018-317581 30954949 PMC6943247

[B3] Ferrer-Villada T. Navarro-Polanco R. A. Rodríguez-Menchaca A. A. Benavides-Haro D. E. Sánchez-Chapula J. A. (2006). Inhibition of Cardiac HERG Potassium Channels by Antidepressant Maprotiline. Eur. J. Pharmacol. 531 (1-3), 1–8. 10.1016/j.ejphar.2005.10.036 16423345

[B4] Fu X. Liu M. Qu S. Ma J. Zhang Y. Shi T. (2018). Exosomal microRNA-32-5p Induces Multidrug Resistance in Hepatocellular Carcinoma via the PI3K/Akt Pathway. J. Exp. Clin. Cancer Res. 37 (1), 52. 10.1186/s13046-018-0677-7 29530052 PMC5846230

[B5] Gao L. Wang X. Tang Y. Huang S. Hu C.-A. A. Teng Y. (2017). FGF19/FGFR4 Signaling Contributes to the Resistance of Hepatocellular Carcinoma to Sorafenib. J. Exp. Clin. Cancer Res. 36 (1), 8. 10.1186/s13046-016-0478-9 28069043 PMC5223586

[B6] Gunduz O. Topuz R. D. Karadag C. H. Ulugol A. (2016). Analysis of the Anti-allodynic Effects of Combination of a Synthetic Cannabinoid and a Selective Noradrenaline Re-uptake Inhibitor in Nerve Injury-Induced Neuropathic Mice. Eur. J. Pain. 20 (3), 465–471. 10.1002/ejp.752 26206340

[B7] Hong P. Liu Q.-W. Xie Y. Zhang Q.-H. Liao L. He Q.-Y. (2020). Echinatin Suppresses Esophageal Cancer Tumor Growth and Invasion through Inducing AKT/mTOR-dependent Autophagy and Apoptosis. Cell Death Dis. 11 (7), 524. 10.1038/s41419-020-2730-7 32655130 PMC7354992

[B8] Hu H.-F. Xu W. W. Li Y.-J. He Y. Zhang W.-X. Liao L. (2021). Anti-allergic Drug Azelastine Suppresses colon Tumorigenesis by Directly Targeting ARF1 to Inhibit IQGAP1-ERK-Drp1-Mediated Mitochondrial Fission. Theranostics 11 (4), 1828–1844. 10.7150/thno.48698 33408784 PMC7778598

[B9] Huang H. Chen J. Ding C. M. Jin X. Jia Z. M. Peng J. (2018). Lnc RNA NR 2F1‐ AS 1 Regulates Hepatocellular Carcinoma Oxaliplatin Resistance by Targeting ABCC 1 via miR‐363. J. Cel Mol Med. 22 (6), 3238–3245. 10.1111/jcmm.13605 PMC5980138 29602203

[B10] Kainov Y. Favorskaya I. Delektorskaya V. Chemeris G. Komelkov A. Zhuravskaya A. (2014). CRABP1 Provides High Malignancy of Transformed Mesenchymal Cells and Contributes to the Pathogenesis of Mesenchymal and Neuroendocrine Tumors. Cell Cycle. 13 (10), 1530–1539. 10.4161/cc.28475 24626200 PMC4050158

[B11] Kellenberger E. Muller P. Schalon C. Bret G. Foata N. Rognan D. (2006). Sc-PDB: an Annotated Database of Druggable Binding Sites from the Protein Data Bank. J. Chem. Inf. Model. 46 (2), 717–727. 10.1021/ci050372x 16563002

[B12] Kim G.-H. Kan S.-Y. Kang H. Lee S. Ko H. M. Kim J. H. (2019). Ursolic Acid Suppresses Cholesterol Biosynthesis and Exerts Anti-cancer Effects in Hepatocellular Carcinoma Cells. Int. J. Mol. Sci. 20 (19), 4767. 10.3390/ijms20194767 PMC6802365 31561416

[B13] Kim Y.-S. Lee Y.-M. Oh T.-I. Shin D. Kim G.-H. Kan S.-Y. (2018). Emodin Sensitizes Hepatocellular Carcinoma Cells to the Anti-cancer Effect of Sorafenib through Suppression of Cholesterol Metabolism. Int. J. Mol. Sci. 19 (10), 3127. 10.3390/ijms19103127 PMC6213641 30321984

[B14] Kugel S. Hingorani S. R. (2020). Cholesterol Biosynthesis Influences Subtype Specificity and Plasticity in Pancreas Cancer. Cancer Cell 38 (4), 443–445. 10.1016/j.ccell.2020.09.010 33049206 PMC9976615

[B15] Li B. Xu W. W. Han L. Chan K. T. Tsao S. W. Lee N. P. Y. (2017). MicroRNA-377 Suppresses Initiation and Progression of Esophageal Cancer by Inhibiting CD133 and VEGF. Oncogene 36 (28), 3986–4000. 10.1038/onc.2017.29 28288140 PMC5511242

[B16] Li J. Hao Q. Cao W. Vadgama J. V. Wu Y. (2018). Celecoxib in Breast Cancer Prevention and Therapy. Cancer. Manag. Res. 10, 4653–4667. 10.2147/cmar.S178567 30464589 PMC6208493

[B17] Lin Y.-L. Persaud S. D. Nhieu J. Wei L.-N. (2017). Cellular Retinoic Acid-Binding Protein 1 Modulates Stem Cell Proliferation to Affect Learning and Memory in Male Mice. Endocrinology 158 (9), 3004–3014. 10.1210/en.2017-00353 28911165 PMC5659671

[B18] Liu S. Gao Y. Zhang L. Yin Y. Zhang W. (2020). Rspo1/Rspo3‐LGR4 Signaling Inhibits Hepatic Cholesterol Synthesis through the AMPKα‐SREBP2 Pathway. FASEB J. 34 (11), 14946–14959. 10.1096/fj.202001234R 32926477 PMC8016451

[B19] Liu X. Wu Y. Zhou Z. Huang M. Deng W. Wang Y. (2019). Celecoxib Inhibits the Epithelial-To-Mesenchymal Transition in Bladder Cancer via the miRNA-145/TGFBR2/Smad3 axis. Int. J. Mol. Med. 44 (2), 683–693. 10.3892/ijmm.2019.4241 31198976 PMC6605707

[B20] Lu S. Yao Y. Xu G. Zhou C. Zhang Y. Sun J. (2018). CD24 Regulates Sorafenib Resistance via Activating Autophagy in Hepatocellular Carcinoma. Cel Death Dis. 9 (6), 646. 10.1038/s41419-018-0681-z PMC5974417 29844385

[B21] Mahipal A. Kommalapati A. Mehta R. Kim R. D. (2019). “Molecular-Targeted Therapies in Hepatocellular Carcinoma,” in Hepatocellular Carcinoma: Translational Precision Medicine Approaches. Editor Hoshida Y. (Cham (CH): Humana Press). 32078262

[B22] Mir N. Jayachandran A. Dhungel B. Shrestha R. Steel J. C. (2017). Epithelial-to-Mesenchymal Transition: A Mediator of Sorafenib Resistance in Advanced Hepatocellular Carcinoma. Curr. Cancer. Drug. Targets. 17 (8), 698–706. 10.2174/1568009617666170427104356 28460616

[B23] Park S. W. Nhieu J. Persaud S. D. Miller M. C. Xia Y. Lin Y.-W. (2019). A New Regulatory Mechanism for Raf Kinase Activation, Retinoic Acid-Bound Crabp1. Sci. Rep. 9 (1), 10929. 10.1038/s41598-019-47354-7 31358819 PMC6662813

[B24] Persaud S. D. Lin Y.-W. Wu C.-Y. Kagechika H. Wei L.-N. (2013). Cellular Retinoic Acid Binding Protein I Mediates Rapid Non-canonical Activation of ERK1/2 by All-Trans Retinoic Acid. Cell Signal. 25 (1), 19–25. 10.1016/j.cellsig.2012.09.002 22982089 PMC3508141

[B25] Persaud S. D. Park S. W. Ishigami-Yuasa M. Koyano-Nakagawa N. Kagechika H. Wei L.-N. (2016). All Trans-retinoic Acid Analogs Promote Cancer Cell Apoptosis through Non-genomic Crabp1 Mediating ERK1/2 Phosphorylation. Sci. Rep. 6, 22396. 10.1038/srep22396 26935534 PMC4776112

[B26] Rafiee L. Hajhashemi V. Javanmard S. H. (2016). Maprotiline Inhibits LPS-Induced Expression of Adhesion Molecules (ICAM-1 and VCAM-1) in Human Endothelial Cells. Res. Pharm. Sci. 11 (2), 138–144. 27168753 PMC4852658

[B27] Riscal R. Skuli N. Simon M. C. (2019). Even Cancer Cells Watch Their Cholesterol!. Mol. Cel 76 (2), 220–231. 10.1016/j.molcel.2019.09.008 PMC7225778 31586545

[B28] Ruiz de Galarreta M. Bresnahan E. Molina-Sánchez P. Lindblad K. E. Maier B. Sia D. (2019). β-Catenin Activation Promotes Immune Escape and Resistance to Anti-PD-1 Therapy in Hepatocellular Carcinoma. Cancer Discov. 9 (8), 1124–1141. 10.1158/2159-8290.Cd-19-0074 31186238 PMC6677618

[B29] Silvente-Poirot S. Poirot M. (2014). Cholesterol and Cancer, in the Balance. Science 343 (6178), 1445–1446. 10.1126/science.1252787 24675946

[B30] Tong Y. Jiao Q. Liu Y. Lv J. Wang R. Zhu L. (2018). Maprotiline Prevents Monocrotaline-Induced Pulmonary Arterial Hypertension in Rats. Front. Pharmacol. 9, 1032. 10.3389/fphar.2018.01032 30298002 PMC6160570

[B31] Trott O. Olson A. J. (2009). AutoDock Vina: Improving the Speed and Accuracy of Docking with a New Scoring Function, Efficient Optimization, and Multithreading. J. Comput. Chem. 31 (2), NA. 10.1002/jcc.21334 PMC3041641 19499576

[B32] Wai Ling Khoo T. S. Rehman A. Olynyk J. K. (2019). “Tyrosine Kinase Inhibitors in the Treatment of Hepatocellular Carcinoma,” in Hepatocellular Carcinoma. Editor Tirnitz-Parker J.E.E. (Brisbane (AU): Codon Publications). 31664806

[B33] Xiang D.-M. Sun W. Zhou T. Zhang C. Cheng Z. Li S.-C. (2019). Oncofetal HLF Transactivates C-Jun to Promote Hepatocellular Carcinoma Development and Sorafenib Resistance. Gut 68 (10), 1858–1871. 10.1136/gutjnl-2018-317440 31118247

[B34] Xu W. W. Huang Z. H. Liao L. Zhang Q. H. Li J. Q. Zheng C. C. (2020). Direct Targeting of CREB1 with Imperatorin Inhibits TGF β 2‐ERK Signaling to Suppress Esophageal Cancer Metastasis. Adv. Sci. 7 (16), 2000925. 10.1002/advs.202000925 PMC7435243 32832354

[B35] Xu W. W. Zheng C.-C. Huang Y.-N. Chen W.-Y. Yang Q.-S. Ren J.-Y. (2018). Synephrine Hydrochloride Suppresses Esophageal Cancer Tumor Growth and Metastatic Potential through Inhibition of Galectin-3-AKT/ERK Signaling. J. Agric. Food Chem. 66 (35), 9248–9258. 10.1021/acs.jafc.8b04020 30113849

[B36] Xu W. W. Zheng C.-C. Zuo Q. Li J.-Q. Hong P. Qin Y.-R. (2021). Genome-wide Identification of Key Regulatory lncRNAs in Esophageal Cancer Metastasis. Sig Transduct Target. Ther. 6 (1), 88. 10.1038/s41392-021-00476-9 PMC7910292 33637684

[B37] Yan A. Jia Z. Qiao C. Wang M. Ding X. (2020). Cholesterol Metabolism in Drug-resistant Cancer (Review). Int. J. Oncol. 57 (5), 1103–1115. 10.3892/ijo.2020.5124 33491740

[B38] Yang P. M. Hong Y. H. Hsu K. C. Liu T. P. (2019). p38α/S1P/SREBP2 Activation by the SAM-Competitive EZH2 Inhibitor GSK343 Limits its Anticancer Activity but Creates a Druggable Vulnerability in Hepatocellular Carcinoma. Am. J. Cancer Res. 9 (10), 2120–2139. 10.3892/etm.2021.9854 31720078 PMC6834481

[B39] Zhang X. Chen J. Cheng C. Li P. Cai F. Xu H. (2020). Aspirin Potentiates Celecoxib-Induced Growth Inhibition and Apoptosis in Human Non-small Cell Lung Cancer by Targeting GRP78 Activity. Ther. Adv. Med. Oncol. 12, 175883592094797. 10.1177/1758835920947976 PMC7502795 32994805

[B40] Zuo C. Hong Y. Qiu X. Yang D. Liu N. Sheng X. (2018). Celecoxib Suppresses Proliferation and Metastasis of Pancreatic Cancer Cells by Down-Regulating STAT3/NF-kB and L1CAM Activities. Pancreatology 18 (3), 328–333. 10.1016/j.pan.2018.02.006 29525378

